# Complete Genome Sequence of *Rhodococcus equi* Phage Nova53

**DOI:** 10.17912/micropub.biology.002046

**Published:** 2026-06-17

**Authors:** Riley E. Cornwell, Isabella R. DeJonge, Mike H. Liang, Abby N. Puzemis, Alea L. Casipit, Abbie L. Clement, Seth J. Cooper, Debora B. Ferreira, Gabby J. Halliwill, Jonathan D. Koetje, Garry D. Sprick, Savannah J. Wallace, Ella M. Woolworth, Myles D. Radersma, John T. Wertz, Randall J. DeJong

**Affiliations:** 1 Biology, Calvin University, Grand Rapids, Michigan, United States

## Abstract

Nova53 is a novel bacteriophage with siphovirus morphology isolated from soil using *Rhodococcus equi* NRRL B-16538 as host. Nova53 has a genome of 137,941 base pairs that putatively encode 250 proteins and 31 tRNAs. Based on gene content, Nova53 is assigned to cluster CG that, to date, consists of only two other phages.

**Figure 1. TEM image, plaques, and genome comparison of first 40kb of Nova53:** Negative stain (1% uranyl acetate) transmission electron microscopy revealed siphoviridae morphology (a) of Nova53; scale bar equals 200 nm. In standard plaque assays (b), Nova53 produced tiny (0.5 mm), circular plaques. (c) Alignment of left-end of Nova53 genome with the other two members of Cluster CG, Dorin and Francesca. The genome is represented by the ruler, in kilo base pairs, with boxes above and below the ruler representing forward and reverse transcribed genes, respectively, and gene numbers presented within the box. Putative functions of genes are labeled for Nova53; corresponding genes in the other two genomes can be identified by shared color and position.

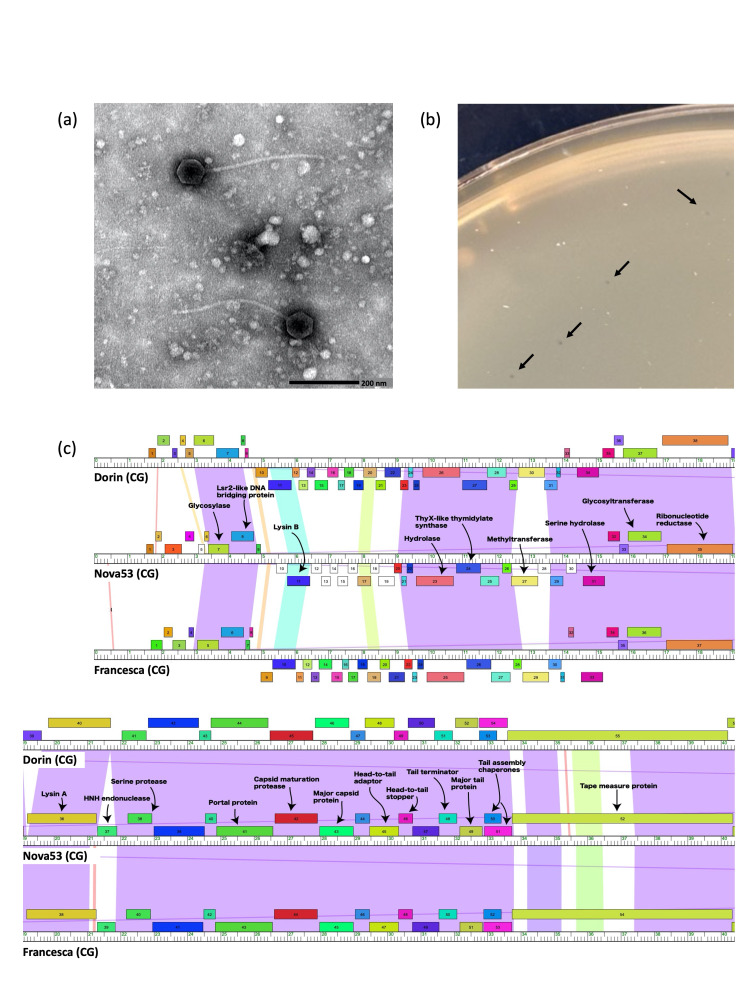



## Description

Actinobacteria *Rhodococcus equi* is a bacterium which opportunistically infects the lungs of domestic animals and immunocompromised humans (Weinstock & Brown, 2002). These bacteria have received minimal attention regarding bacteriophage infection and lysis (Bonilla et al., 2017; Daspit et al., 2025; Radersma et al., 2024; Summer et al., 2011). Calvin University students have isolated and annotated several bacteriophages that infect *R. equi* (Daspit et al., 2025; Radersma et al., 2024). Here we present Nova53, a cluster CG bacteriophage with siphovirus morphology, isolated using *R. equi* NRRL B-16538.

Nova53 was isolated from a soil sample taken from Grand Rapids, Michigan (42.930479 N, 85.58862 W) following standard procedures (Zorawik et al., 2024). The soil sample was washed in PYCa broth and the wash filtered with a 0.22µm syringe-top filter. The filtrate was incubated with the host bacteria for two days at 30˚C with shaking and was then refiltered and plated in PYCa top agar with *R. equi* NRRL B-16538. &nbsp;Nova53 formed tiny (<0.5 mm) circular, mostly clear plaques (Figure 1b), and after three rounds of plaque purification a high-titer lysate was prepared. Negative stain (1% uranyl acetate) transmission electron microscopy revealed a siphovirus morphology (Figure 1a).

Phage DNA was extracted from the lysate using a Qiagen DNeasy kit, and a DNA library was prepared for sequencing using a NEBNext Ultra II-FS kit. Then 1.6 million 100-base single end reads of the genome were obtained via an Illumina NextSeq 1000 (XLEAP-P1 kit). Raw reads were trimmed with cutadapt 4.7 (using the option: –nextseq-trim 30) and filtered with skewer 0.2.2 (using the options: -q 20 -Q 30 -n -l 50) prior to assembly (Jiang et al., 2014; Martin, 2011). Genome assembly was achieved using Unicycler v0.5.1 and Consed v29 with 1111-fold shotgun coverage (Gordon et al., 1998; Russell, 2018; Wick et al., 2017).

Analysis and annotation of the Nova53 genome was completed using the following: DNA Master v5.23.6 (Pope & Jacobs-Sera, 2018), Glimmer v3.02 (Kelley et al., 2012), and Genemark v2.5 (Besemer et al., 2001) to first identify open reading frames; Phamerator (Actino_draft database v626) (Cresawn et al., 2011), BLASTp v.2.14.1 (Actinobacteriophage and NCBI non-redundant protein databases) (McGinnis & Madden, 2004), HHPred (PDB, UniProt, Pfam-A v.37, and NCBI v.3.19 databases) (Söding et al., 2005); DeepTMHMM v1.0.24 (Hallgren et al., 2022) to support protein functions; Aragorn (Laslett & Canback, 2004) and tRNAscanSE v2.0 (Lowe & Eddy, 1997) to identify tRNAs; and PECAAN v20221109 (Rinehart et al., 2016) to compile final genomic annotations. Default settings were used for all software.

The genome of Nova53 is 137,941 base pairs, has 250 identifiable protein-coding genes, and is assigned to the CG cluster of actinobacteriophages ( https://phagesdb.org ) based on gene content similarity (GCS) of at least 35% (Pope et al., 2017). This cluster includes two other phages isolated using *R. equi* , Francesca and Dorin (Daspit et al., 2025). The three genomes have similar GC content of 48-48.5% and have terminal repeats that are similar in length (4720-5222 bp) but differ in gene content (Figure 1c). Francesa and Dorin share 91% GCS with one another, but share 66% GCS with Nova53. A high number of Nova53 genes (62) do not have homologs within the current actinobacteriophage database, including a number in the first 15 kbp before the structural genes (Figure 1c). The Nova53 genome shares most of its 31 tRNAs with Francesca and Dorin in similar order. Nova53 contains many of the expected genes in bacteriophages, including terminase, portal, major capsid protein, lysin A, major tail protein, tape measure protein, and minor tail protein. The genome also includes identifiable genes for head-to-tail adapters and stoppers, tail terminators, lysin B, holin, HNH endonuclease, and tail assembly chaperones, though it lacks identifiable genes for capsid maturation protease, scaffolding proteins, and tail fibers. A considerable number of nucleic acid replication and recombination enzymes, including DNA helicase, DNA primase, DnaE-like DNA polymerase III alpha, RecA-like DNA recombinase, Holliday Junction resolvase, and RuvC-like resolvase follow the structural genes and are shared with Dorin and Francesca. Neither integrase nor immunity repressor functions were discovered in the genome, suggesting a lytic lifestyle.

**Data availability.** Annotated genome sequence can be accessed for Nova 53 at GenBank accession PV876963 . Sequence reads are deposited at NCBI under SRA accession number SRX31207624 .
